# Transcriptomic characterization of cold acclimation in larval zebrafish

**DOI:** 10.1186/1471-2164-14-612

**Published:** 2013-09-11

**Authors:** Yong Long, Guili Song, Junjun Yan, Xiaozhen He, Qing Li, Zongbin Cui

**Affiliations:** 1 The Key Laboratory of Aquatic Biodiversity and Conservation, Institute of Hydrobiology, Chinese Academy of Sciences, Wuhan, Hubei, PR China; 2 University of the Chinese Academy of Sciences, Beijing, PR China

**Keywords:** Cold stress, Transcriptomic analysis, Zebrafish, RNA-seq, Gene expression

## Abstract

**Background:**

Temperature is one of key environmental parameters that affect the whole life of fishes and an increasing number of studies have been directed towards understanding the mechanisms of cold acclimation in fish. However, the adaptation of larvae to cold stress and the cold-specific transcriptional alterations in fish larvae remain largely unknown. In this study, we characterized the development of cold-tolerance in zebrafish larvae and investigated the transcriptional profiles under cold stress using RNA-seq.

**Results:**

Pre-exposure of 96 hpf zebrafish larvae to cold stress (16°C) for 24 h significantly increased their survival rates under severe cold stress (12°C). RNA-seq generated 272 million raw reads from six sequencing libraries and about 92% of the processed reads were mapped to the reference genome of zebrafish. Differential expression analysis identified 1,431 up- and 399 down-regulated genes. Gene ontology enrichment analysis of cold-induced genes revealed that RNA splicing, ribosome biogenesis and protein catabolic process were the most highly overrepresented biological processes. Spliceosome, proteasome, eukaryotic ribosome biogenesis and RNA transport were the most highly enriched pathways for genes up-regulated by cold stress. Moreover, alternative splicing of 197 genes and promoter switching of 64 genes were found to be regulated by cold stress. A shorter isoform of *stk16* that lacks 67 amino acids at the N-terminus was specifically generated by skipping the second exon in cold-treated larvae. Alternative promoter usage was detected for *per3* gene under cold stress, which leading to a highly up-regulated transcript encoding a truncated protein lacking the C-terminal domains.

**Conclusions:**

These findings indicate that zebrafish larvae possess the ability to build cold-tolerance under mild low temperature and transcriptional and post-transcriptional regulations are extensively involved in this acclimation process.

## Background

Water temperature was suggested to be the master factor that determines nearly all life activities of most fishes, including development, growth, reproduction, metabolism, behavior and geographical distribution [1-3]. Like other ectotherms, fishes may encounter a wide range of daily and seasonal temperature variations in their habitats and deleterious consequences will occur if water temperature exceeds the species-specific thermal tolerance range [2,4]. It was reported that most of fish kills in nature are caused by exposure to low temperatures [5]. Thus, the ability to cope with cold stress is quite important for the survival of fishes under natural conditions. Furthermore, a large number of commercially important aquaculture species such as tilapia (*Oreochromis niloticus*), milkfish (*Chanos chanos*) and red sea bream (*Pagrus major*) are sensitive to cold stress and mass mortality is often caused by winter cold fronts [6-8]. Therefore, it is of great significance for both scientific researches and fisheries to investigate the mechanisms underlying cold-tolerance of fish.

Since the middle of the 20th century, an increasing number of studies were performed to characterize the acclimation responses of fishes to cold stress. It was revealed that fishes can gradually establish cold adaptive phenotypes through extensive biochemical, metabolic and physiological regulations [9,10]. Well-defined biochemical and physiological acclimations include producing temperature-specific isozymes [9], altering the content of membrane lipid and the degree of fatty acid unsaturation [11], recruiting different muscle fiber types [12], synthesizing molecular chaperones [13], and altering mitochondrial densities and their properties [14]. Moreover, microarray techniques have been widely used to profile gene expressions in fishes exposed to short- or long-term cold stresses during the past decade. Researchers have characterized the transcriptional responses elicited by cold stress in fishes such as common carp (*Cyprinus carpio*) [15], zebrafish (*Danio rerio*) [16-18], channel catfish (*Ictalurus punctatus*) [19], annual killifish (*Austrofundulus limnaeus*) [20], coral reef fish (*Pomacentrus moluccensis*) [21] and rainbow trout (*Oncorhynchus mykiss*) [22]. These investigations have revealed a large number of cold-regulated genes involved in a variety of biological processes that are associated with acclimation to both daily and seasonal low temperatures.

It is well documented that the cold-tolerance of fishes is mainly determined by genetics, thermal history and developmental stages and fishes at larvae stages are more sensitive to cold injury than adults [4,23,24]. Despite the importance of larvae survival for fishery production and the stability of wild population, there is little information about the acclimation of fish larvae to cold stress. In some insects, a phenomenon named “rapid cold hardening (RCH)” can be evoked by a mild cold exposure for a period of minutes or a few hours, which is important for protecting insects from cold injury at both organismal and cellular levels [25]. The RCH response has been described in both chill-sensitive and chill-tolerant insects and is remarkable for its rapid induction [26]. However, it is unknown whether fish larvae possess such a rapid acclimation response to cold stress and the transcriptional and posttranscriptional regulations in fish larvae exposed to cold stress remain to be defined.

Zebrafish is widely used as a research model for multiple disciplines including developmental biology, genetics, physiology, toxicology and environmental genomics. Abundant biological information and genetic resources have been accumulated for this species, e.g., the complete genome sequence and comprehensive annotations, which markedly facilitate investigations using high-throughput techniques such as microarray and RNA-seq. Moreover, zebrafish is a tropical eurythermal fish that might encounter a wide range of both daily and seasonal temperature fluctuations. Zebrafish acclimated to 20°C demonstrated critical thermal maxima (CTMax) and critical thermal minima (CTMin) of 39.2°C and 6.2°C, respectively [2,27]. We have characterized the gene expression profiles in zebrafish larvae exposed to low or high temperature stress [18], but the post-transcriptional regulations remain unclear due to the limitations of microarray technique. RNA-Seq is a recently developed approach to determine the transcriptomic profiles using deep-sequencing technologies, which exhibits some advantages over microarray such as the independency of existing genomic sequence, high sensitivity and accuracy, digital expression and the ability to distinguish transcript isoforms [28].

This study aims to characterize the ability of zebrafish larvae to build cold-tolerance after exposed to a mild low temperature and investigate the transcriptional responses elicited by cold stress. We found that 96 hpf zebrafish larvae exposed to a mild low temperature (16°C) for 24 h exhibited a significant increase in survival rates under further severe cold stress at 12°C. Transcriptional responses behind the formation of cold-tolerance were characterized using RNA-seq. The sequencing reads were mapped to zebrafish genome sequence and assembled into transcripts. A total of 23,693 genes were found to be expressed and differential expression analysis identified 1,431 up- and 399 down-regulated genes. Enrichment analysis of Gene Ontology (GO) terms and Kyoto Encyclopedia of Genes and Genomes (KEGG) pathways have revealed that RNA splicing, protein catabolic process and ribosome biogenesis were the most enriched GO terms and that spliceosome, proteosome and ribosome biogenesis in eukaryotes were the most overrepresented pathways among cold-induced genes. Furthermore, cold stress led to differential splicing of 197 genes and promoter switching of 64 genes. Obviously, these findings would be very important for further understanding the intracellular signaling mechanisms of cold stress in fish.

## Results

### Development of cold resistance in zebrafish larvae

**Figure 1:**
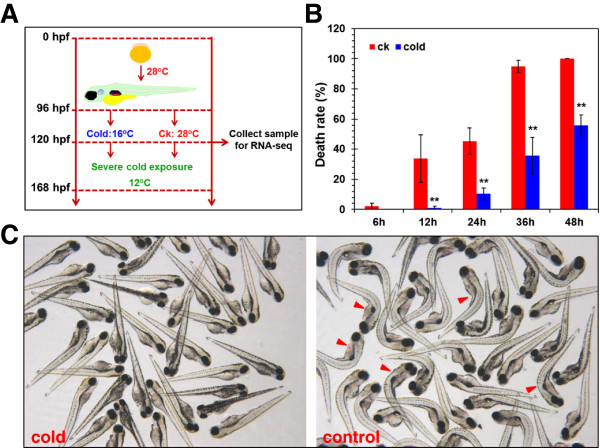
**Establishment of cold resistance in zebrafish larvae after pre-exposure to cold stress. (A)** Flowchart of cold exposure. Zebrafish embryos were incubated at 28°C from fertilization to 96 hpf. Larvae at 96 hpf were exposed to 16°C for 24 h and the controls were maintained at 28°C. Samples for RNA-seq were collected at 120 hpf. The pre-treated and control larvae were further exposed to 12°C for 6, 12, 24, 36 and 48 h. The time scales were shown on the left. **(B)** Death rates of pre-treated and control (ck) larvae exposed to 12°C for different times. Data was shown as mean ± standard deviation (n = 4). “**” above error bars indicate *p* < 0.01. **(C)** Images of pre-treated and control larvae exposed to 12°C for 36 h. Images were taken under a stereomicroscope from Zeiss with a color CCD camera. Red arrowheads indicate representative dead larvae.

To address whether zebrafish larvae are able to build cold-resistance under a mild low temperature, 96 hpf larvae were incubated at 16°C for 24 h followed by severe cold exposure at 12°C and the controls were maintained at 28°C before severe cold exposure at 12°C (Figure 1A). Larvae at 96 hpf were used to perform the experiment because larvae at this stage need not to be fed and are less sensitive to cold stress than the earlier stage embryos [18]. As shown in Figure 1B, the death rates of the pre-treated larvae are significantly lower than those of the control at 12, 24, 36 and 48 h (p < 0.01 in all cases). The death rates after severe cold exposure for 36 and 48 h reached 94.93% and 100% in the control, but were 35.71 and 55.93% in the pre-treated group. After severe cold exposure at 12°C for 36 h, the survival larvae in experimental group were normal in morphology; however, most of the control larvae were dead and displayed an obvious body curvature (Figure 1C). These findings suggest that zebrafish larvae developed the resistance against severe cold stress after a pre-exposure at 16°C.

### Mapping of RNA-seq reads to zebrafish genome

To reveal the cold acclimation-associated transcriptional responses in larval zebrafish, three biological replicates of both control and cold-treated samples were subjected to total RNA extraction and RNA-seq analysis. High-throughput sequencing generated 19.48-26.51 million (M) pairs of raw reads for each of the sample and more than 96% of the raw reads passed quality filtering (Q > 20 and length ≥ 25 bp) (Table 1). After the quality filtering, paired reads were extracted and mapped to the zebrafish genome using TopHat. A total of 37.18-50.20 M reads were processed by TopHat and the percent of mapped reads were quite similar among different samples (91.77-92.56%). The number of total mapping events generated by TopHat was 45.55-57.91 M and the number of potential splices was 5.27-5.32 M, representing 11.54-15.33% of the total alignment. The ratio of unique map to the total alignment was 75.25-78.50%; however, the ratio of uniquely mapped reads to the total number of processed reads was 98.73-99.74% (Table 1).

**Table 1:** Statistics for the filtering and mapping of reads

**Sample name**	**ck1**	**ck2**	**ck3**	**cold1**	**cold2**	**cold3**
Total reads (M)	19.80x2	24.72x2	19.48x2	26.51x2	21.07x2	24.57x2
Good reads (M)	38.05	47.76	37.62	50.92	40.74	47.56
% Good reads	96.09	96.61	96.58	96.06	96.67	96.80
Processed reads (M)	37.52	47.22	37.18	50.20	40.25	46.95
Mapped reads (M)	34.85	43. 40	34.38	46.06	37.26	43.34
% Mapped	92.89	91.90	92.47	91.77	92.56	92.33
Total alignment (M)	45.55	56.44	45.57	57.91	47.28	55.32
Total potential splices (M)	5.27	5.32	5.27	5.32	5.28	5.27
% Reads mapped to junction	15.13	12.26	15.33	11.54	14.16	12.16
Unique mapping (M)	34.41	43.00	34.29	45.46	36.82	43.02
% Unique mapping	75.54	76.17	75.25	78.50	77.87	77.75
% Uniquely mapped reads	98.73	99.07	99.74	98.70	98.83	99.24

% unique mapping indicates the ratio of unique mapping to the total alignment.

### Gene expression detected by RNA-seq

The mapping data generated by TopHat was processed by Cufflinks toolkits for transcript assembly and differential expression analysis. The abundance of gene transcripts was expressed as FPKM (Fragments per kilobase of transcript per million fragments mapped) [29]. To identify expressed genes, background coverage for intergenic regions was calculated as well. As shown in Additional file 1, the mean background coverage was 0.029 FPKM and a significant decrease in frequency occurred at 0.1 FPKM. Therefore, genes with a mean abundance > 0.1 FPKM in either the control or cold-treated samples were regarded as being expressed and a total of 23,693 expressed genes were detected in this study (Table 2).

**Table 2:** Statistics of genes regulated by cold stress

**Classification**	**Number**
Detected genes	23693
Up-regulated genes	1431
Down-regulated genes	399
Differentially spliced genes	197
Genes with promoter-switching	64

**Figure 2:**
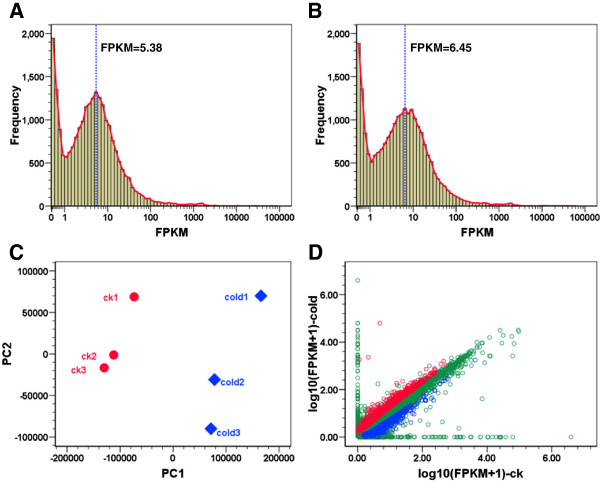
**Bioinformatic analysis of RNA-seq data. (A** and **B)** Distribution of FPKM values for genes expressed in the control **(A)** and cold-treated **(B)** zebrafish larvae. The red interpolation line denotes a bimodal distribution of the frequency of FPKM. The blue dashed line indicates the FPKM value for the second peak of frequency. **(C)** Principle component analysis of gene expression in the control (ck) and cold-treated samples. **(D)** Correlation of gene expression between the control and cold-treated group. The up- and down-regulated genes were shown in red and blue, respectively. Genes not regulated by cold treatment were shown in green.

As shown in Figure 2A and B, the abundance of most genes was less than 100 FPKM and both the control and cold-treated samples demonstrated a bimodal frequency curve of FPKM values. The first peak of the frequency curves was located at 0.1 FPKM and the second peak was located at 5.38 and 6.45 FPKM for the control and cold-treated samples, respectively. The trough between the two peaks was near 1 FPKM and the baselines of the second frequency peak range from 1 to 20 FPKM for both groups (Figure 2A and B). Therefore, genes were classified as low (FPKM ≤ 1), medium (1 < FPKM ≤ 20) and high abundance (FPKM > 20) according to their FPKM values. The largest portion (more than 60%) of expressed genes belongs to the medium-abundance, followed by low-abundance genes (nearly 23%, Additional file 2). A smaller number of medium-abundance genes and a larger number of high-abundance genes were detected in cold-treated samples than those in the control, suggesting that the expression of many genes was induced in cold-treated larvae.

Principle component analysis (PCA) was performed to characterize the overall effects of cold stress on gene expression. The results of PCA indicate that 78.52% of the variations in gene expression can be explained by the first two principle components (PC). The inter-group and intra-group variations in gene expression were captured by PC1 and PC2 respectively (Figure 2C). PCA projections of the control and cold-treated samples in the principle component space exhibited a clear discrepancy, indicating that significant variations in gene expression occurred after cold exposure.

The differentially expressed genes between the control and cold-treated groups were displayed in Figure 2D. Genes with fold change ≥ 2, p-value < 0.05 and q-value < 0.05 were considered to be differentially expressed. The numbers of up- and down-regulated genes after cold exposure were 1,431 and 399 (Table 2). Genes regulated by cold stress were listed in Additional file 3. Genes such as *il12a* (interleukin 12a) and *sp8b* (sp8 transcription factor b) were found to be specifically expressed in cold-treated larvae. Genes *ptgr1* (prostaglandin reductase 1), *irg1l* (immunoresponsive gene 1, like) and *mmp13a* (matrix metalloproteinase 13a) were among the most highly up-regulated genes under cold stress, while *mep1b* (meprin A, beta), *rh50* (Rh50-like protein) and *nr5a5* (nuclear receptor subfamily 5, group A, member 5) were representative genes inhibited by cold stress. A comparison with our previous study [18] revealed a significant overlap between the up-regulated genes detected by microarray and RNA-seq (Additional file 4). Since larvae exposed to cold stress for different time periods were used in these studies, considerable discrepancy in gene expression was also found.

### Validation of RNA-seq data by quantitative real time PCR (qPCR)

**Figure 3:**
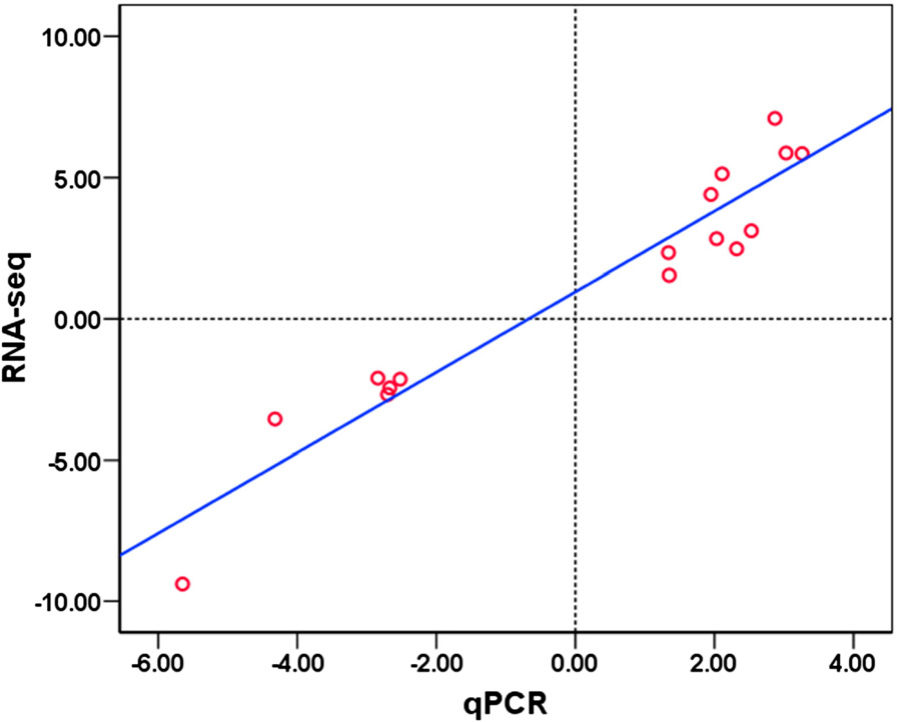
**Validation of RNA-seq data using qPCR.** Fold changes of gene expression detected by RNA-seq were plotted against the data of qPCR. The reference line indicates the linear relationship between the results of RNA-seq and qPCR.

**Figure 4:**
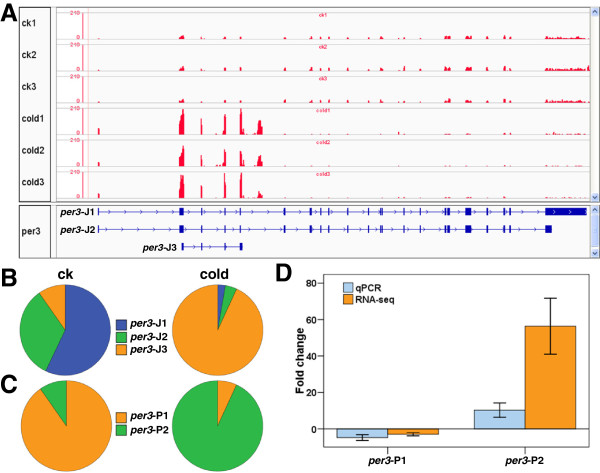
**Alternative promoter usage of *per3 *under cold stress. (A)** Read coverage at *per3* locus. The top panel shows the read coverage of each sample at *per3* locus and the bottom panel indicates the structure of *per3* transcripts. **(B)** The relative abundance of *per3* isoforms under cold stress. **(C)** Promoter switching of *per3* under cold stress. **(D)** qPCR validation of the alternative promoter usage for *per3*. Error bars indicate standard deviation (n = 3).

To validate the expression profiles from RNA-seq analysis, relative mRNA levels for 15 genes were measured by qPCR. The expression data for these genes and transcript isoforms detected by RNA-seq and qPCR are displayed in Table 3 and Figure 3. Expression of these genes and transcripts except *cry-dash*, were confirmed by qPCR. The down-regulation of *per3* upon cold stress was detected by probes targeting *per3-J1* and *per3-J2* (transcripts of per3-P1 in Table 3 and Figure 4) in our previous microarray study [18]. However, RNA-seq explicitly revealed the down-regulation of *per3-J1* and *per3-J2* and the up-regulation of *per3-J3* (transcript of per3-P2 in Table 3 and Figure 4) in this study. The data from qPCR and RNA-seq exhibited excellent agreement on both up- and down-regulated genes. The correlation between microarray and qPCR data was analyzed by Spearman’s rho test and a highly statistical significance [r (17) = 0.938, p = 0.000001] was observed.

**Table 3:** Comparisons between RNA-seq data and qPCR results

**Gene symbol**	**Gene name**	**Fold change**	
		**qPCR**	**RNA-seq**
*brf2*	BRF2, subunit of RNA polymerase III transcription initiation factor, BRF1-like	3.3	5.9
*per2*	Period homolog 2	3.0	5.9
*nrf1*	Nuclear respiratory factor 1	2.3	2.5
*fosl1a*	FOS-like antigen 1a	2.1	5.1
*gabpa*	GA-binding protein transcription factor, alpha subunit	2.0	2.8
*per1b*	Period homolog 1b	2.0	4.4
*Per3-*total	Period homolog 3	2.9	5.8
*per3-*P1	Period homolog 3	−4.8	−3.0
*per3-*P2	Period homolog 3	10.0	56.3
*fos*	v-fos FBJ murine osteosarcoma viral oncogene homolog	2.5	3.1
*stk16-j1*	Serine/threonine kinase 16	1.4	1.5
*cry-dash*	Cryptochrome DASH	1.3	2.3
*her8a*	Hairy-related 8a	−2.5	−2.1
*setd7*	SET domain containing (lysine methyltransferase) 7	−2.7	−2.5
*sirt3*	Sirtuin (silent mating type information regulation 2 homolog) 3	−2.7	−2.7
*stc1l*	Stanniocalcin 1, like	−2.8	−2.1
*nr5a5*	Nuclear receptor subfamily 5, group A, member 5	−5.7	−9.4

### Gene ontology (GO) enrichment analysis of genes regulated by cold stress

**Figure 5:**
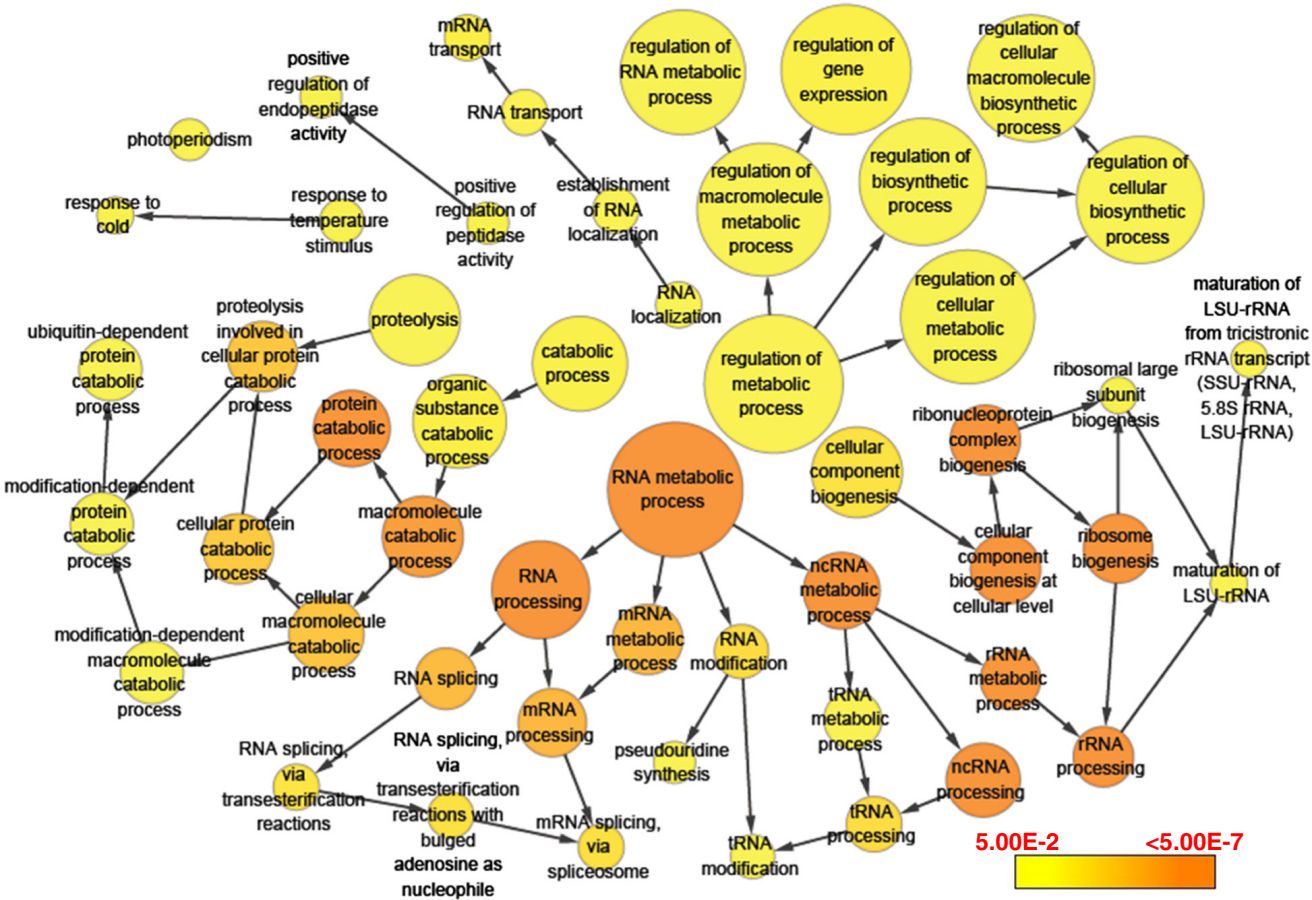
**GO enrichment analysis for genes up-regulated by cold stress.** The size of circles is proportional to the number of genes associated with the GO term. The arrows represent the relationship between parent–child terms. The color scale indicates corrected p-value of enrichment analysis.

GO enrichment analysis was performed to reveal the biological processes overrepresented under cold stress. As shown in Figure 5, five main functional categories including RNA metabolic process, cellular component biogenesis, regulation of metabolic process, catabolic process and RNA localization were significantly enriched from genes up-regulated by cold stress. More specific terms of these enriched categories include RNA splicing, ribosome biogenesis and protein catabolic process (Figure 5).

**Figure 6:**
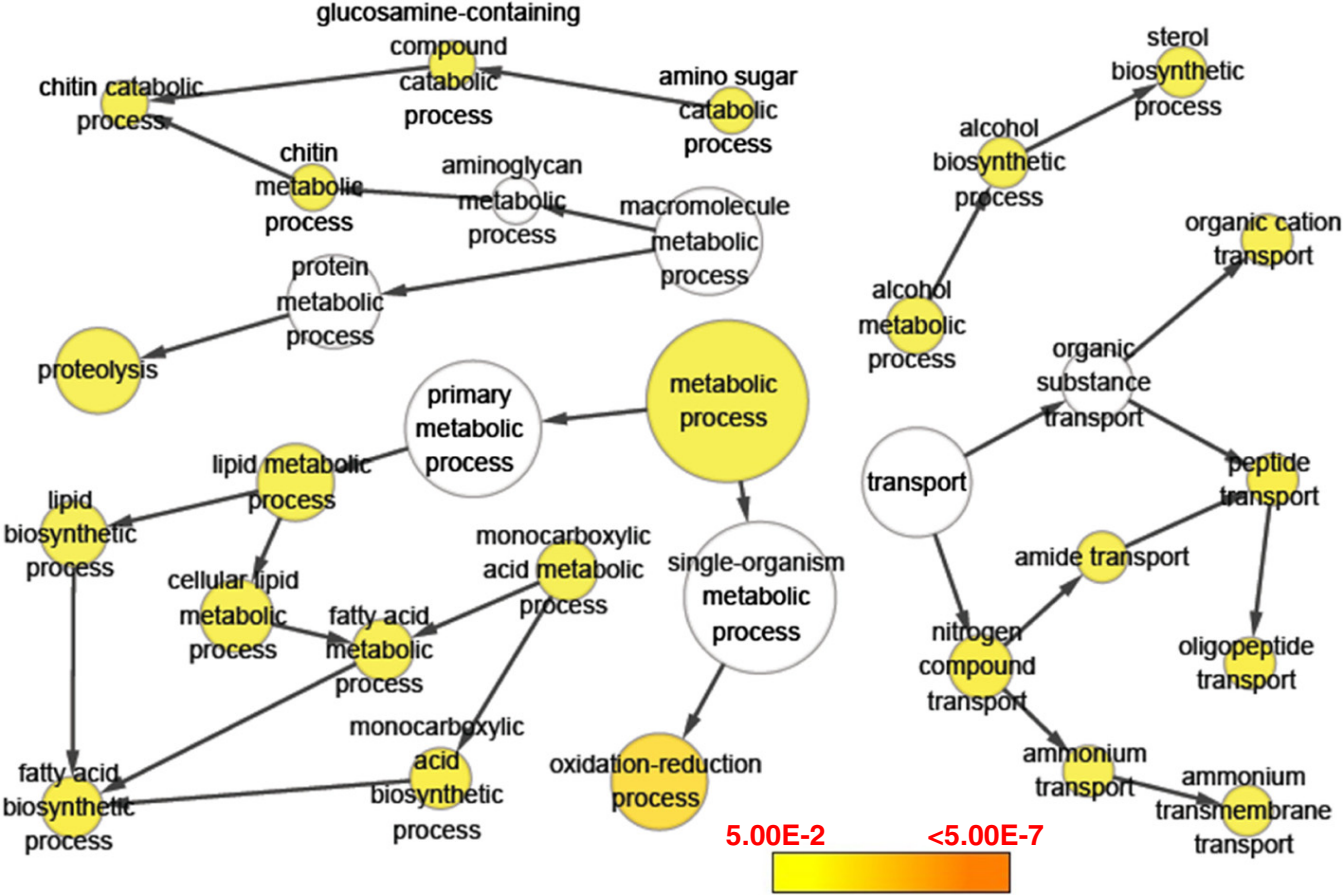
**GO enrichment analysis for genes down-regulated by cold stress.** The size of circles is proportional to the number of genes associated with the GO term. The arrows represent the relationship between parent–child terms. The color scale indicates the corrected p-value of enrichment analysis.

The most specific GO terms overrepresented by cold-inhibited genes include oxidation-reduction process, fatty acid biosynthetic process, proteolysis, sterol biosynthetic process, ammonium transmembrane transport, oligopeptide transport, organic cation transport and chitin catabolic process (Figure 6). Genes associated with the enriched GO terms were shown in Additional file 5. Some of these enriched GO terms from this study such as RNA processing, ubiquitin-dependent protein catabolic process and proteolysis were previously found to be overrepresented in cold-regulated genes [15,18]. The results of GO enrichment analysis indicate that multiple biological processes are involved in the establishment of cold resistance in zebrafish larvae.

### KEGG pathway enrichment analysis of genes regulated by cold stress

**Figure 7:**
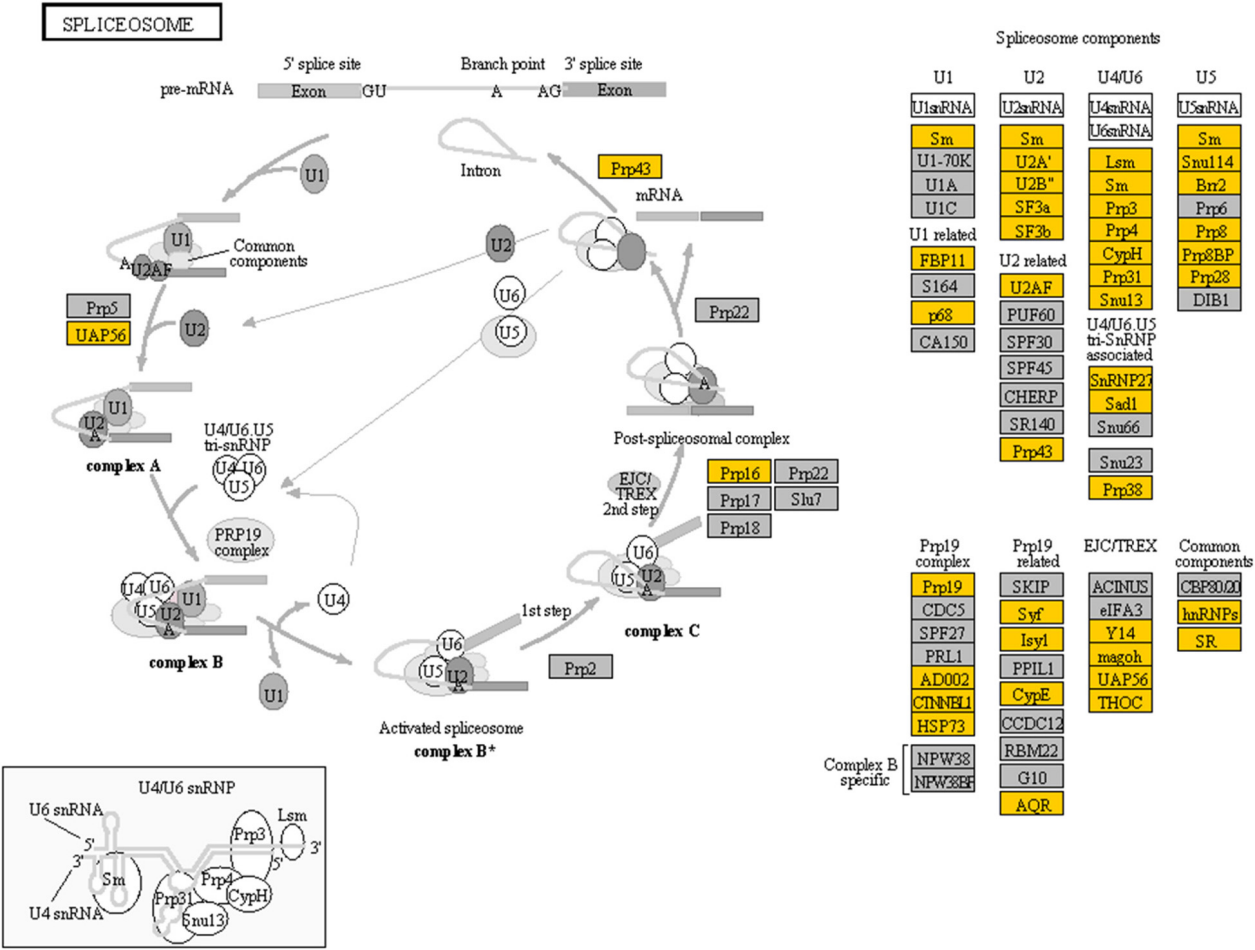
**Cold-induced genes associated with the spliceosome pathway.** Gene expression value was mapped to the reference pathway using the KegArray. Up-regulated genes are shown in yellow.

The results of KEGG pathway enrichment analysis were displayed in Additional file 6. A total of 61 pathways were overrepresented in genes up-regulated by cold stress. Spliceosome was the most significantly enriched pathway, in which 43.33% (52) of the associated genes were up-regulated by cold stress. The components of spliceosome include the U1, U2, U4, U5 and U6 small nuclear ribonucleoprotein particles (snRNPs), each of which consists of a specific small nuclear RNA (snRNA), a common set of seven Sm/Lsm proteins and variable number of particle-specific proteins [30,31]. We found that the Sm/Lsm genes and nearly all others associated with U2, U4/U6 and U5 were up-regulated by cold stress (Figure 7). Some of the cold-induced genes have been shown to play essential roles in splicing. For example, U2 specific splicing factors SF3a (splicing factor 3a, subunit 2) and SF3b (splicing factor 3B subunit 3-like) are involved in stabilizing the U2/BS (Branch site) duplex [31], and members of the DExD/H-box family of RNA unwindases/RNPases such as Brr2 (pre-mRNA-splicing helicase BRR2), Prp28 (DEAD box polypeptide 23) and Prp43 (DEAH box polypeptide 15) are responsible for driving rearrangements of the spliceosome’s RNA-RNA and RNA-protein networks required for splicing [31,32]. In addition, some genes involved in the non-snRNPs complexes such as Prp19 complex were up-regulated by cold stress as well (Figure 7).

**Figure 8:**
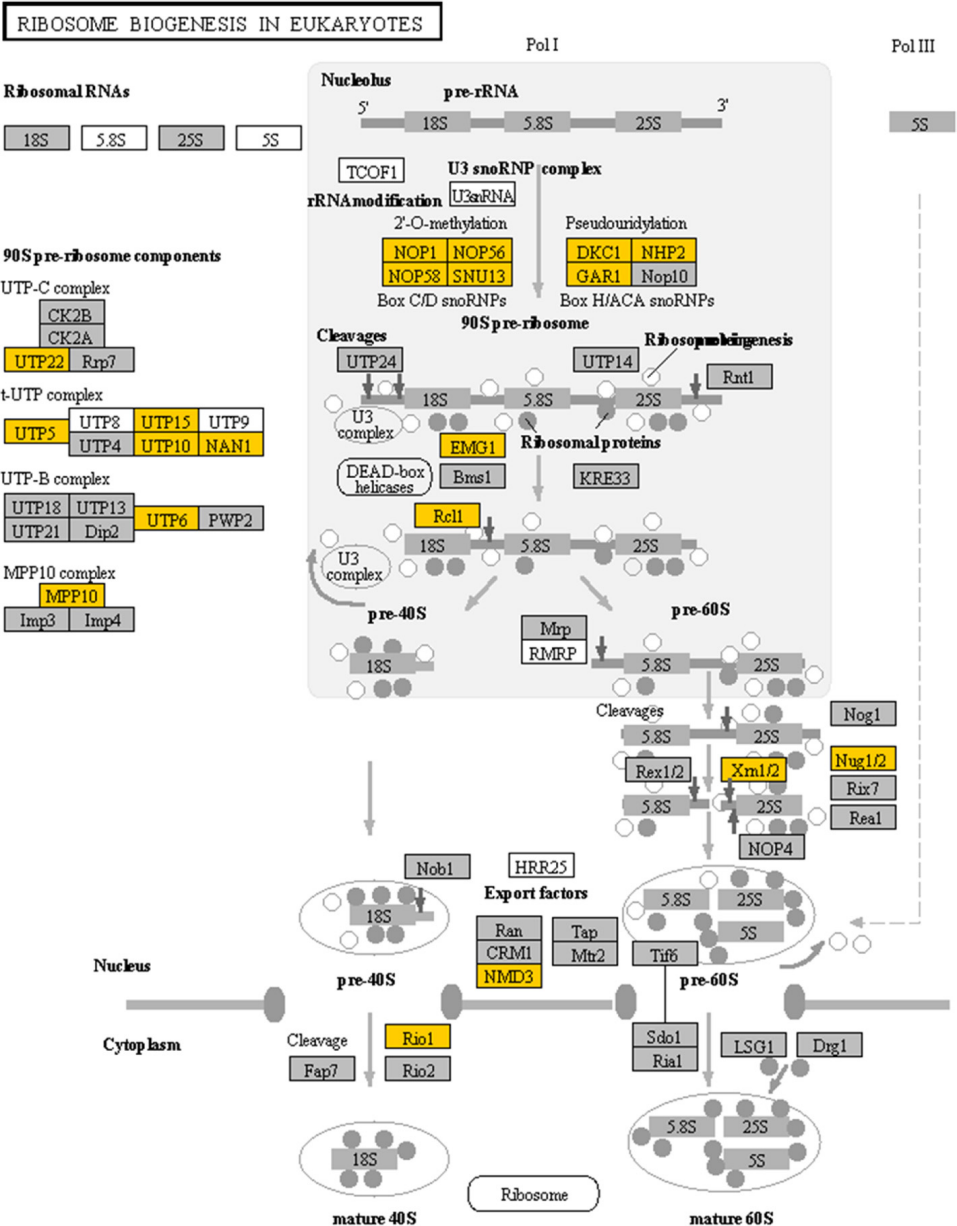
**Cold-induced genes associated with the ribosome biogenesis in eukaryotes pathway.** Gene expression value was mapped to the reference pathway using the KegArray. Up-regulated genes are shown in yellow.

Ribosome biogenesis in eukaryotes is another highly represented pathway with 30.88% (21) of the associated genes up-regulated by cold stress (Additional file 6 and Figure 8). The process of ribosome biogenesis involves the maturation of ribosomal rRNAs and their assembly into ribosomal subunits [33]. Among the multiple steps during the ribosomal subunit biogenesis, cold-induced genes mainly function in the rRNA modification process. The 2′-O-methylation and pseudouridylation are the most prevalent modifications of rRNAs. The modified nucleotides are important for the conformation and stabilization of RNA and the activity of ribosome in translation [33]. Among the cold-induced genes associated with ribosome biogenesis, Nop1 (fibrillarin), Nop56 (NOP56 ribonucleoprotein homolog), Nop58 (NOP58 ribonucleoprotein homolog, yeast) and Snu13 (NHP2 non-histone chromosome protein 2-like 1b, yeast) are associated with 2′-O-methylation. Dkc1 (dyskeratosis congenita 1, dyskerin), Nhp2 (NHP2 ribonucleoprotein homolog, yeast) and Gar1 (GAR1 ribonucleoprotein homolog, yeast) are related to pseudouridylation of rRNAs. Utp5 (WD repeat domain 43), Utp10 (HEAT repeat-containing protein 1-like), Utp15 (U3 small nucleolar ribonucleoprotein, homolog) and Nan1 (WD repeat domain 75) are building blocks of the t-UTP complex, which is required for the subsequent assembly of other 90S pre-ribosome components [34]. Additionally, genes associated with other 90S pre-ribosome components and the maturation of 40S and 60S subunits were up-regulated by cold stress (Figure 8).

Other representative pathways regulated by cold stress include RNA transport, pyrimidine metabolism, RNA polymerase, purine metabolism, mRNA surveillance pathway, herpes simplex infection, protein processing in endoplasmic reticulum and circadian rhythm. Moreover, many pathways involved in signal transduction such as MAPK signaling, p53 signaling, ErbB signaling, Wnt signaling and mTOR signaling pathways were enriched from cold-induced genes. Furthermore, pathways associated with cell communication such as tight junction and gap junction were overrepresented in cold-induced genes. These findings indicate the importance of signal transduction and cell communication in the acclimation of zebrafish larvae to cold stress.

Pathways overrepresented by cold-inhibited genes include steroid biosynthesis, peroxisome, drug metabolism, amino sugar and nucleotide sugar metabolism, inositol phosphate metabolism, cardiac muscle contraction (Additional file 6). Since most of these pathways are associated with metabolism, cold stress appears to suppress the basal metabolism of zebrafish larvae.

### Differential splicing under cold stress

**Figure 9:**
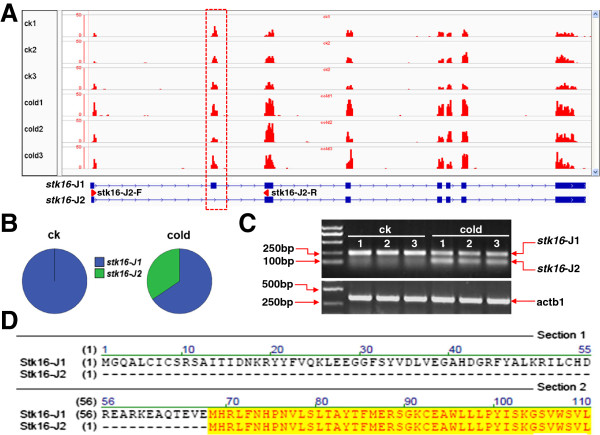
**Differential splicing of *stk16 *under cold stress. (A)** Read coverage at *stk16* locus. The top panel shows the read coverage of each sample at *sk16* locus and the bottom panel indicates the structure of *stk16* transcripts. The red arrowheads represent relative positions of primers for RT-PCR. The dashed red box indicates the unchanged coverage of exon 2 under cold stress. **(B)** RT-PCR analysis to reveal the cold-specificity of *stk16-J2*. Stk16-J2-F and stk16-J2-R primers were displayed in **(A)**. **(C)** The relative abundance of *stk16* isoforms under cold stress. **(D)** Partial peptide sequence alignment of Stk16-J1 and Stk16-J2. The identical amino acids are shown in yellow.

In addition to differentially expressed genes, Cuffdiff can identify genes with differential splicing and promoter switching under different conditions [35]. A total of 197 genes were found to be differentially spliced in zebrafish larvae after exposure to cold stress (Additional file 7). Results of functional clustering reveal that these differentially spliced genes are classified into eight functional clusters in which regulation of transcription is the most highly represented biological process (Additional file 8). The expression of *stk16* (Ser/Thr kinase 16) was characterized as an example of posttranscriptional regulation induced by cold stress. Zebrafish *stk16* gene is located on chromosome 1, which can generate one transcript as predicted in Ensembl (http://www.ensembl.org/). This transcript encodes a peptide of 306 amino acids (designated as *stk16-J1*). Cufflincks assembled a new transcript (*stk16-J2*) lacking the second exon in *stk16-J1* and the coverage of all other exons was markedly increased in cold-treated samples when compared to the controls (Figure 9A). The results of differential expression analysis revealed that *stk16-J2* was specifically expressed in larvae exposed to cold stress (Figure 9B). RT-PCR analysis confirmed the specificity of *stk16-J2* in cold-treated samples (Figure 9C). The skipping of exon 2 led to a truncated peptide lacking the N-terminal 67 amino acids (Figure 9D).

### Alternative promoter usage under cold stress

When a gene is transcribed from multiple transcription start sites (TSSs) due to the alternative usage of promoters, Cuffdiff will allocate them into different TSS groups by adding up the expression levels of isoforms, which reflect the promoter preference between conditions [35]. In this study, putative promoter switching was found in 64 genes under cold stress (Additional file 9). Among these genes, zebrafish *per3* gave rise to three transcripts including *per3-*J1, *per3-*J2 *and per3-*J3 (Figure 4A). *Per3-*J1 and *per3-*J2 share the same TSS (*per3*-P1) and open reading frame, while *per3-*J3 uses a different TSS (*per3*-P2) to generate a short transcript (Figure 4A). The expression level of *per3-*J3 was significantly induced, but those of *per3-*J1 and *per3-*J2 were inhibited by cold stress (Figure 4A and B). Moreover, the *per3-*J3 became the dominant transcript under cold stress, indicating *per3*-P2 is a cold- preferred promoter (Figure 4C). The up-regulation of *per3*-P2 under cold stress was further validated by qPCR analysis (Figure 4D).

Since zebrafish *per3* was reported to be a circadian gene [36], we further characterized the expression pattern of *per3* transcripts during a 24 h light–dark cycle. Zebrafish larvae were kept under light–dark cycle from fertilization and exposed to 16°C from 96 hpf to 144 hpf. As shown in Additional file 10, the overall expression of *per3*-P1 transcripts under 28°C peaked at 12:00 am and dropped to trough at 12:00 pm; however, when exposed to cold stress the level of *per3*-P1 transcripts decreased at first and peaked at 8:00 pm. The level of *per3*-J3 was very low and no diurnal expression patterns were found in control larvae maintained at 28°C. Upon cold exposure, the expression level of *per3*-J3 continuously increased and the peak was found at 8:00 pm (Additional file 10). These results indicate that cold stress can lead to the alteration in the circadian rhythm of longer *per3* transcripts *per3*-J1 and *per3*-J2, and induce the shorter transcript *per3*-J3 showing no diurnal expression patterns under normal temperature.

## Discussion

It has been suggested that genetic background, thermal history and developmental stages are the most important factors determining the cold-tolerance of fish [2,4,24]. Most of the previous studies of cold acclimation in fish have focused on long-term and especially seasonal adaptive responses in juveniles or adults. It was recently shown that the thermal experience of fish at embryonic stages can have dramatic and persistent effects on thermal acclimation capacity of adults [37,38]. However, the adaptive responses of fish larvae to cold stress and the transcriptional alterations during these processes remain largely unknown. In this study, we demonstrated that exposure of 96 hpf zebrafish larvae to 16°C for 24 h led to a significant increase in survival rates of developing larvae under further severe cold stress at 12°C, indicating that zebrafish larvae possess a RCH-like ability to build cold-tolerance under mild low temperature. To our knowledge, this is the first evidence indicating the existence of RCH-like responses in fish. In insects, the elevated body levels of low molecular cryoprotectants such as glucose, trehalose and glycerol were suggested to be the biochemical basis of the RCH [26,39]. In fishes, increases in the ratio of unsaturated fatty acids and plasma lactate concentration [7], up-regulated mitochondria biogenesis [10], biosynthesis of isoenzymes [9] and regulation of muscle fiber types [40] were found to be associated with long-term cold acclimation. To further address the molecular mechanisms underlying the establishment of cold-tolerance in fish larvae, we investigated the transcriptional responses during the establishment of cold-tolerance using RNA-seq. A total of 1431 up- and 399 down-regulated genes were identified in 23693 expressed genes of zebrafish larvae. These differentially expressed genes are involved in many crucial processes and pathways such as RNA splicing, protein catabolic process, ribosome biogenesis, spliceosome, proteosome and ribosome biogenesis. Additionally, the alternative splicing of 197 genes and promoter switching of 64 genes were found to be specifically regulated by cold stress. Obviously, findings of the study are of great importance for further investigation of the cold-specific signaling networks in fish.

Previous studies have shown that a very large number of genes are involved in the acclimation of fish to cold stress. A systematic microarray study of carp tissues exposed to low temperature for 22 days identified 3461 cold-regulated cDNAs [15]. Many of cold-induced genes were found to be regulated by both short- and long-term cold stress. Representatives of these genes include cold-inducible RNA binding protein (*cirbp*), high-mobility group proteins (*hmgb1a*, *hmgb1b*), ribonucleoproteins involved in mRNA processing and splicing (*snrpd3*, *snrpa1*, *prpf8* and *sf3a2*) and translation initiation factors (*eif1axa*, *eif1axb*, *eief2a* and *denr*). Thus, these genes can be used as potential molecular markers for characterization of the cold acclimation responses in different fish species. Moreover, many of the highly overrepresented functional categories and pathways of cold-induced genes from this study are overlapped with those found in all the analyzed tissues of carp exposed to cold stress [15], indicating the importance of these processes or pathways in the establishment of cold-tolerance. Since RNA processing and ribosome biogenesis processes are directly associated with gene expression, the alterations of these biological processes are consistent with the notion that more up-regulated genes are usually found than down-regulated genes in transcriptomic analysis of fishes exposed to cold stress [15,19]. Furthermore, protein catabolic processes such as the ubiquitin-dependent protein catabolism were often found to be induced by cold exposure, suggesting the degradation and modification of unfolded or misfolded proteins under low temperature stress.

The sensing and intracellular transduction of stress signals is critical for the adaptation and survival of organisms under various environmental stresses. Information about the mechanisms whereby fish cells sense the cold signal and trigger the intracellular responses is extremely scarce. In this study, mitogen-activated protein kinase (MAPK), p53 and peroxisome proliferator-activated receptor (PPAR) signaling were found to be the most highly enriched signal transduction pathways under cold stress. The canonical MAPK signaling pathway is a three-component signal cascade in which an activated MAPK kinase kinase (MAPKKK or MEKK) activates a MAPK kinase (MAPKK or MEK), which then activates an extracellular signal-regulated kinase MAPK or ERK [41]. It has been shown that MAPK pathway is involved in intracellular responses to diverse environmental stresses including cold, heat, reactive oxygen species, UV, desiccation and pathogen attack [42,43]. The p53 signaling pathway can be induced by stress signals such as DNA damage, hypoxia, heat and oxidative stress and its activation often leads to cell cycle arrest, cellular senescence or apoptosis [44-46]. The PPAR signaling pathway is mainly involved in the protection of cells against oxidative stress and apoptosis [47,48]. Overrepresentation of these signaling pathways among cold-induced genes suggests their importance in the transduction of cold signals and the establishment of cold-tolerance in fish. Since protein phosphorylation is extensively involved in the activation of these signaling pathways, characterizing the phosphorylation of intracellular proteins upon cold stress would provide further insights into the networks for sensing and transduction of cold signal.

Alternative splicing events such as exon skipping, alternative usage of 5′/3′ splice sites and intron retention may lead to changes of the amino- or carboxy-terminus, in-frame addition/removal of a functional unit and insertion of premature termination codon, thus contributing to both transcriptomic and proteomic diversities [49]. Accumulating evidence indicates that alternative splicing is important for adaptation responses to a wide range of stress conditions [50-54]. Stress-associated alternative splicing is mainly described for genes encoding the protein kinases, transcription factors, splicing regulators and pathogen-resistance factors, and the alternative splicing under stress conditions usually leads to changes in the subcellular localization, binding properties, and activity or stability of the resulting proteins [49]. In this study, alternative splicing of 197 genes and promoter switching of 64 genes were found to be regulated by cold stress. The generation of a short *stk16* splicing isoform under cold stress was selected for validation of transcriptome analysis. It is known that STK16 functions as a transcriptional co-activator in the expression regulation of vascular endothelial growth factor (VEGF) [55]. However, the functional significance of alternative splicing of zebrafish *stk16* under cold stress remains unclear. Moreover, promoter switching was confirmed by molecular analysis of *per3* transcripts and a short isoform containing 4 exons was found to be highly up-regulated under cold stress. It has been demonstrated that *per3* is involved in multiple processes such as circadian rhythm, sleep, cancer, cell proliferation and apotosis [56-58]. Further investigations are needed to reveal the functions of cold-specific splicing variants and transcripts in the cold acclimation of zebrafish.

Nearly all eukaryotic cells possess self-sustained circadian clocks that couple endogenous biochemical, physiological and behavioral rhythms with environmental changes [59]. The effects of temperature on circadian clocks have been well established. Except for day/night cycles, temperature changes serve as a zeitgeber for circadian clocks and temperature cycles of as little as 2°C are sufficient to entrain the clock [60-62]. Temperature changes in physiological range can influence the amplitude of circadian transcriptional rhythms but not alter the period length of circadian cycles (temperature compensation) [61,62]. Out of the range of temperature compensation, the clock stops running and arrests at a certain phase [63]. In plants, cold-responsive pathways and cold tolerance are intimately associated with circadian clocks [64-66]. In zebrafish, many core clock genes such as nuclear receptors (*nr1d1*, *nr1d2a*, *nr1d2b*, *nr1d4a* and *nr1d4b*), period homologs (*per1a*, *per1b*, *per2* and *per3*) and cryptochromes (*cry1a*, *cry1b*, *cry2* and *cry2b*) were induced by cold stress (Additional file 3), suggesting the involvement of circadian clocks in cold acclimation. Furthermore, we identified a shorter isoform of *per3* gene highly up-regulated by cold stress (Additional file 10). This short isoform lacks the functional PAS (Per-Arnt-Sim) domain and the period circadian-like C-terminal domain and is similar to a small interference peptide (siPEP), which serves as a dominant-negative to interfere the activities of corresponding transcription factors in plants under cold stress [67]. Further investigations are needed to address if the cold-specific transcription of *per3* is related to temperature compensation of circadian clock and cold acclimation in zebrafish.

Cells adapt to stresses or changing environmental conditions as a result of alterations in gene expression at multiple levels including transcriptional, post-transcriptional and translational regulations [68]. Although a large number of genes were found to be up-regulated at transcriptional level upon cold stress, the correlation between gene transcription and the proteomic landscape remains to be characterized. The activity of proteins is dependent on their correct folding and only correctly folded proteins have the long-term stability in crowded biological environments and are able to interact selectively with their natural partners [69]. The folding of proteins is a temperature-dependent process and the hydrophobic effect driving protein folding decreases with dropping temperature [70,71]; therefore, the folding rate of proteins decreases at reduced temperature [72]. The landscape of protein folding in living cells can affect gene expression at transcriptional level, so the up-regulated mRNA level of some genes and alternative splicing events under cold stress could be a compensating measure for the reduced protein folding rate. Further investigations are needed to address the correlation between cold stress-induced transcriptomic and proteomic responses in fish.

## Conclusions

This study has revealed the existence of cold acclimation in zebrafish larvae. Further transcriptomic analysis has uncovered many cold-regulated genes encoding proteins that are key components of some crucial biological processes and signaling pathways such as RNA splicing, ribosome eukaryotic ribosome biogenesis, protein catabolism, spliceosome, proteasome and RNA transport. Additionally, a large number of alternative splicing and promoter switching events were identified to be specifically regulated during the establishment of cold acclimation in zebrafish. These findings have provided novel clues for further investigation of the molecular mechanisms underlying the cold acclimation in zebrafish.

## Methods

### Animals and cold exposure

The animal protocol for this study was approved by the Institutional Animal Care and Use Committee of Institute of Hydrobiology (Approval ID: Y21304501). Maintenance of adult zebrafish and incubation of embryos were performed as previously described [18,73]. Biochemical incubators (HWS-150, Shanghai Jinghong laboratory instrument Co., Ltd.) were used for temperature control and incubation of embryos. During cold exposure, zebrafish larvae at 96 hpf were transferred immediately into dishes (50 larvae per dish) containing culture medium preconditioned at 16°C and incubated for 24 h (Figure 1). The controls were maintained at 28°C. No embryonic mortality was observed during the treatment. After cold exposure, the dishes were placed on ice for 5 min to anesthetize the larvae and samples were collected and subjected to RNA extraction for preparation of RNA library followed by RNA-seq.

To evaluate the ability of zebrafish larvae to build cold resistance after pre-exposure to 16°C, pre-treated and control larvae at 120 hpf were further exposed to severe cold stress at 12°C for 6, 12, 24, 36 and 48 h, respectively (Figure 1A). The larvae were then maintained at 28°C for another 24 h. Larvae displaying no heart beat and no response to touch were regarded as dead and removed. To avoid the side effects of light period on gene expression and cold resistance, embryos and larvae were kept in dark throughout the experiment.

### Library construction and high-throughput sequencing

Zebrafish larvae in the same dish were collected at 120 hpf for RNA extraction (Figure 1A). Total RNA extraction was performed with TRIZOL reagents from Invitrogen following the manufacturer’s instructions. Total RNA contents were measured using the NanoDrop 8000 from Thermo Scientific and the quality of RNA samples was assessed by agarose gel electrophoresis.

RNA library construction was then performed by SinoGenoMax Co., Ltd, Beijing, China (http://www.sinogenomax.com/). Before library construction, the integrity of RNA samples was confirmed using Agilent 2100 Bioanalyzer and 4 μg of total RNA was used for isolation of mRNA with Sera-mag Magnetic Oligo (dT) beads from Illumina. The purified mRNA was fragmented into small pieces (100–400 bp) using divalent cations at 94°C for 5 minutes. Double-stranded cDNA was synthesized using the SuperScript Double-Stranded cDNA Synthesis kit (Invitrogen, Camarillo, CA) with random hexamer primers from Illumina. The synthesized cDNA was subjected to end-repair, phosphorylation, 3′ adenylation and adapter ligation in sequential. After these steps, cDNA fragments ranging from 250 to 350 bp were collected and purified by gel electrophoresis. The purified cDNA template was enriched by PCR amplification and the quality of RNA library was validated in a LightCycler480 (Roche Diagnostics) using an Illumina PhiX174 Control. Three independent biological replicates for both control and cold-treated larvae were used for library construction and RNA-seq analysis. High-throughput sequencing was performed by experts in the Analytical & Testing Center at Institute of Hydrobiology, Chinese Academy of Sciences (http://www.ihb.ac.cn/fxcszx/). Multiplexed libraries were sequenced for 36 bp at both ends using an Illumina Genome Analyzer IIx platform according to the standard Illumina protocols as reported previously [29]. The sequencing data have been deposited in NCBI Sequence Read Archive (SRA, http://www.ncbi.nlm.nih.gov/Traces/sra) and the accession number is SRA062881.

### Bioinformatic analysis of RNA-seq data

The raw reads were trimmed and filtered using PRINSEQ (version 0.19.3) [74]. Low quality (Q < 20) and ambiguous bases (N) were first trimmed from both ends of the reads and the trimmed reads were filtered with Phred quality score (Q ≥ 20 for all bases) and read length (≥ 25 bp). Paired reads were extracted using cmpfastq (http://compbio.brc.iop.kcl.ac.uk/software/cmpfastq.php). Read mapping, transcript assembly and differential expression analysis were performed according to the protocols described previously [35]. Briefly, the preprocessed reads were mapped to the genome sequence of zebrafish (Zv9.68) using TopHat (version 2.0.4) [75] with default parameters except “--segment-mismatches 1” and “--segment-length 18”. The aligned reads were assembled into transcripts using Cufflinks (version 2.0.2) [76] with the following parameters “--frag-bias-correct, --multi-read-correct, --library-type fr-unstranded, --upper-quartile-norm, --total-hits-norm”. The assembled transcripts were merged with the reference annotation (Danio_rerio.Zv9.68.gtf, downloaded from Ensembl) using cuffmerge. Differential expression analysis was performed using cuffdiff with the parameters “--upper-quartile-norm” and “--total-hits-norm”; the merged assembly and the fragment alignments generated by TopHat were used as input files. Calculation of mapping statistics, sorting and indexing of the read alignment files were performed using SAMtools (version 0.1.18) [77]. The mapping and assembling results were viewed via the IGVtools (version 2.1) [78].

### Background estimation

To determine the background in RNA-seq analysis, 20 intergenic regions about 5 kb were randomly selected from each chromosome using IGVtools [78]. The selected intergenic regions were treated as exons and their FPKM values were calculated using cuffdiff as described above.

### Principle component analysis (PCA)

PCA was performed using Arraytrack [79] to elucidate the overall patterns of gene expression in the control and cold-treated samples. FPKM values of all genes identified as expressed were used for the analysis.

### Quantitative real time PCR (qPCR)

qPCR analysis was performed according to the MIQE (Minimum information for publication of quantitative real-time PCR experiments) guidelines to validate the results of RNA-seq. First-strand cDNA for each sample was synthesized from 4 μg of total RNA using random hexamer primer with the RevertAidTM First Strand cDNA Synthesis Kit from Fermentas. The PCR primers were designed using Primer Premier 6.0 software. qPCR was performed in a CFX Connect^TM^ Real-Time PCR Detection System from BioRad. The amplification was carried out in a volume of 20 μL containing 10 μL of 2 × SYBER Green Real Time PCR Master mix from TOYOBO, 2 pmol of each primer and 5 μL of 10 × diluted cDNA templates. Three independent biological replicates of the control and cold-treated groups were included in the analysis and all reactions were carried out in triplicates. The qPCR amplification protocol was 95°C for 1 min, followed by 40 cycles of 95°C for 10 sec, 57-60°C for 30 sec (with plate read) and 72°C for 10 sec. After denaturized at 95°C for 10 sec, the melt curve of PCR product was generated by heating from 65°C to 95°C with 0.5°C increments and 5 sec dwell time, and a plate read at each temperature. The purity of reaction product was confirmed by the observation of a single melt peak. The amplification cycle displaying the first significant increase of the fluorescence signal was defined as threshold cycle and used for quantification (Cq).

Before qPCR analysis, the standard curve of each primer pair was generated by the regression of Cq values and a series of 10-fold cDNA dilutions from the mixture of all samples to be analyzed. The amplification efficiency of primers was calculated from the slope of corresponding standard curve. The sequences and amplification efficiency of primers, the accession number and official name of target genes and the length of amplicons were listed in Additional file 11. *Actb1* was not found to be differentially expressed among samples by RNA-seq and therefore was used as internal reference for the normalization of gene expression. The mean normalized expression of target genes was calculated using the Q-Gene software [80].

### GO and KEGG pathway enrichment analyses

Cytoscape (version 2.8.3) [81] plugins BiNGO (v.2.44) [82] and ClueGO (v.1.5) [83] were used for GO and KEGG pathway enrichment analyses, respectively. All the genes identified in this study were used as reference for the enrichment analysis. Hypergeometric test was used to identify overrepresented GO and KEGG pathway terms with a significance level at 0.05 and Beniamini & Hochberg method was used for the correction of the p-values. The ontology and annotation files for GO enrichment analysis were downloaded from the gene ontology website (http://www.geneontology.org/) at 08.11.2012 and the database used for KEGG pathway enrichment analysis was released on 09.11.2012.

### Functional clustering of differentially spliced genes

The database for annotation, visualization and integrated discovery (DAVID) web software (v6.7, http://david.abcc.ncifcrf.gov/home.jsp) was used for the functional clustering of differentially spliced genes according to GO biological process category [84]. Gene identifier conversion was performed by g:Profiler web software (http://biit.cs.ut.ee/gprofiler/gconvert.cgi) [85].

### Reverse transcription PCR (RT-PCR)

First strand cDNA samples were synthesized from DNaseI (Promega) treated total RNAs as described above. The expected amplicon size and sequences of primers used for this analysis were displayed in Additional file 10. *Actb1* was used as internal control for the success of reverse transcription and PCR amplification. Amplification products were separated by electrophoresis on a 1.5% agarose gel and stained with ethidium bromide.

### Statistical analysis

SPSS 15.0 software for windows was used for statistical analysis. Independent-samples t-test was performed to analyze the significant difference (p < 0.05) in death rates between control and cold-treated larvae after exposure to 12°C. The correlation between the data of RNA-seq and qPCR was analyzed by the Spearman’s rho test.

## Competing interests

The authors declare that they have no competing interests.

## Authors’ contributions

All the authors have read and approved the final manuscript. YL and ZC conceived the study and wrote the manuscript. YL and GS performed the data analysis. YL, YJ, GS and XH performed the experiments. GS and QL provided experimental materials.

## Supplementary Material

Additional file 1 **Distribution of background coverage.** The mean and standard deviation of background FPKM values are shown in the chart. Most (cumulative percent = 95.8) of the background FPKM values are less than 0.1. The blue dashed line indicates the threshold used for identification of expressed genes. Click here for file

Additional file 2 Number of genes expressed with different abundance. Click here for file

Additional file 3 Genes regulated by cold stress. Click here for file

Additional file 4 A comparison between the results of microarray and RNA-seq. Click here for file

Additional file 5 Enriched GO terms for cold-regulated genes. Click here for file

Additional file 6 Enriched pathways for cold-regulated genes. Click here for file

Additional file 7 Genes exhibited differential splicing under cold stress. Click here for file

Additional file 8 Functional clustering of differentially spliced genes. Click here for file

Additional file 9 Genes exhibited alternative promoter usage under cold stress. Click here for file

Additional file 10 **Expression patterns of *per3 *transcripts during a 24 h light−dark cycle.** (A) Expression of *per3*-P1 transcripts (*per3*-J1 and *per3*-J2). (B) Expression of *per3*-J3 (*per3*-P2). Zebrafish larvae were maintained under regular 12 h light−dark cycles from fertilization (light on at 8:00 am, light off at 8:00 pm) and exposed to cold stress (16°C) from 96 hpf to 144 hpf. The mRNA levels were detected using qPCR and the expression level relative to beta-actin was calculated using Q-gene method. Click here for file

Additional file 11 Primers used for qPCR and RT-PCR. Click here for file
