# Supplementary figures and images for: Transcriptomic characterization of cold acclimation in larval zebrafish
Source: BMC Genomics. 2013 Sep 11;14:612. doi: 10.1186/1471-2164-14-612 (PMC3847098; doi:10.1186/1471-2164-14-612)

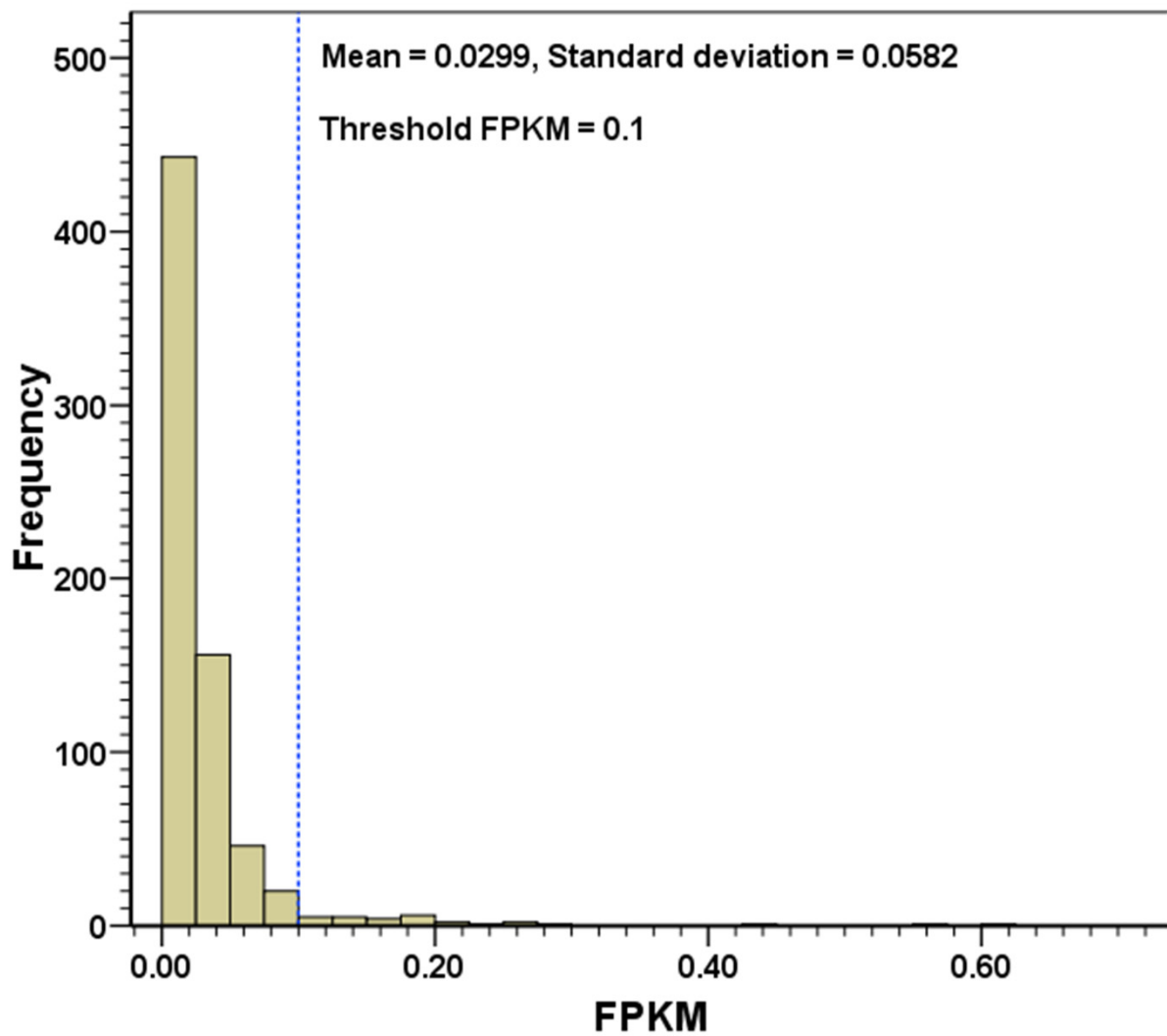

Supplement: Additional file 1 — Distribution of background coverage. The mean and standard deviation of background FPKM values are shown in the chart. Most (cumulative percent = 95.8) of the background FPKM values are less than 0.1. The blue dashed line indicates the threshold used for identification of expressed genes. [file 1471-2164-14-612-S1.pdf]

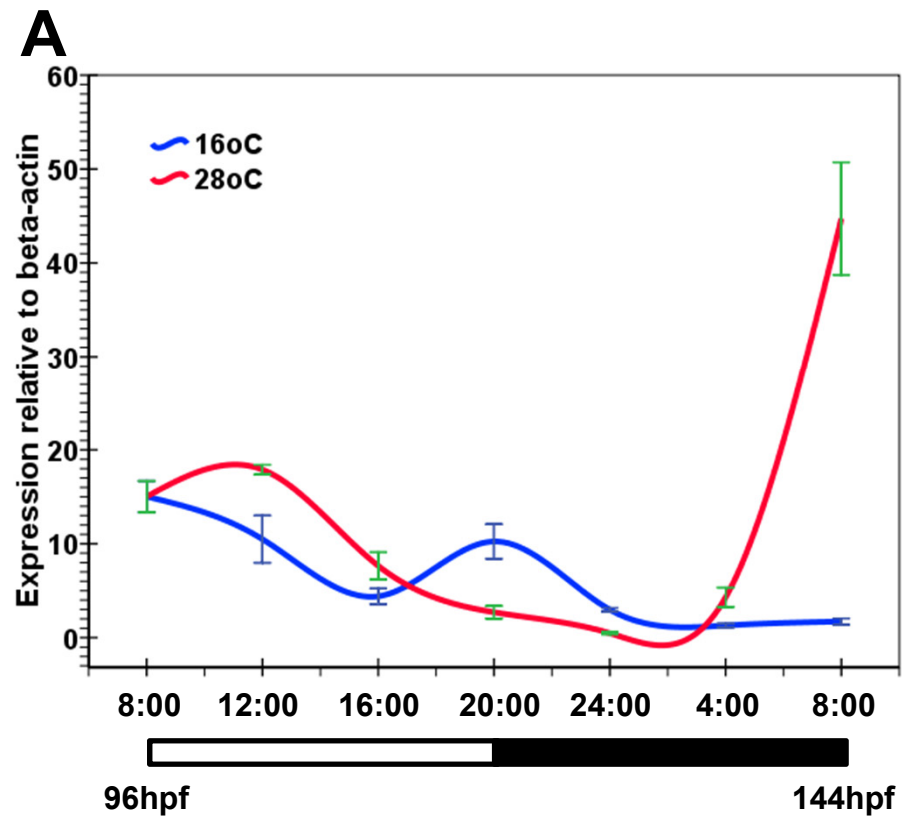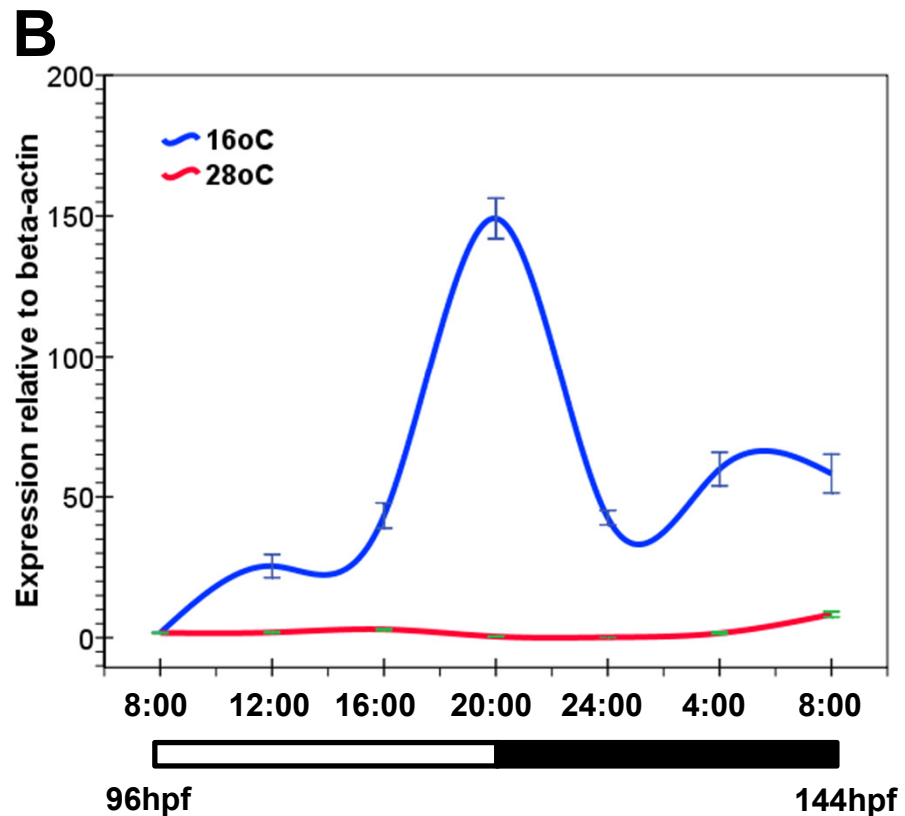

Supplement: Additional file 10 — Expression patterns of per3 transcripts during a 24 h light−dark cycle. (A) Expression of per3-P1 transcripts (per3-J1 and per3-J2). (B) Expression of per3-J3 (per3-P2). Zebrafish larvae were maintained under regular 12 h light−dark cycles from fertilization (light on at 8:00 am, light off at 8:00 pm) and exposed to cold stress (16°C) from 96 hpf to 144 hpf. The mRNA levels were detected using qPCR and the expression level relative to beta-actin was calculated using Q-gene method. [file 1471-2164-14-612-S10.pdf]
